# Hospitals and Paying Patients

**Published:** 1906-03-17

**Authors:** 


### The Hospital.

A Journal of

The Medical Sciences and Hospital Administration.

Vol. XXXIX.—No. 1,016. SATURDAY, MARCH 17, 1906.

### Hospitals and Paying Patients.

Thirty years ago, and once again this week, the *Times* and other papers threw open their columns to correspondents who advocated the establishment of a pay hospital in this country, where members of the middle classes might receive hospital care and treatment for a payment of what was described as a reasonable and inclusive fee. In practice it was found that a payment of a guinea per week represented such a fee in the minds of most of the people who supported the establishment of the Pay Hospital. After three years a sufficient sum was raised to open the Home Hospital, known as Fitzroy House, Fitzroy Square, in 1880. An association was incorporated without the word "limited" under the Companies Acts, and the main object was to give the patients the best nursing and hospital care for an inclusive payment at the lowest cost which would make the institution self-supporting. This Pay Hospital has been continued ever since and has rendered excellent service to the medical profession, and to the patients who have had the advantage of treatment within its walls. It was rebuilt only three years ago, and it now contains thirty beds, an operation theatre, an electric lift and every equipment and facility for the adequate treatment of paying patients.

Why then is it that the Home Hospitals Association has not opened a number of other pay hospitals? Why is it that the minimum rates charged have been increased from three to four guineas a week instead of being reduced to one or two guineas a week as the public demand? The reason is wholly financial, for it has been found in practice that it is not possible to erect and maintain a modern, up to date hospital for the reception of paying patients in any locality adjacent to that in which the majority of the surgeons reside, where patients can be maintained on a self supporting basis at a less cost than four guineas per week. Yet, that the demand exists for cheaper accommodation for patients, who are willing to pay for their treatment, is undoubted, and the demand has increased greatly since the number of surgical patients have multiplied so enormously in recent years. We could have wished, therefore, that the suggestion now made to provide such accommodation had been formulated in some other form than that now put forward, which, as we have shown, is proved by experience to be impracticable for financial reasons.

Our experience in this country in regard to the cost of the provision of pay hospital accommodation is well-nigh identical with that of other countries throughout the world. Our American cousins are deservedly recognised as keen men of business, and, although there are many private hospitals of a costly character in the United States, the many middle-class people get their hospital care through the accommodation provided in pay wings by the larger public hospitals of various kinds. When a member of the middle classes requires an operation or hospital care he at once communicates with one of the large hospitals, and makes arrangements for his admission to a pay ward. The pay wards in private pavilions are kept distinct from the hospital proper, though they are administered and controlled by the managers of the general hospital. The private patients have a separate entrance, and their wards are specially equipped for the purpose and are provided with a separate nursing staff. Each patient for the most part makes his own arrangements with the medical attendant he may select, and the minimum cost per week is usually not much, if at all, less than £5. In some of the private pavilions the patient may pay as much as £15 or £20 per week, but in such a case the accommodation may include a private bathroom and a room for a friend or relative. In a few municipal hospitals provision has been made for the accommodation of relatively poor patients who pay an inclusive charge which will probably range from two to three guineas a week.

In this country, as we have said, financial considerations must be met, or no solution will be found. We have no space to-day to elaborate a scheme of pay wards in connection with the general hospitals and poor-law infirmaries in this country. We may, however, state that such a scheme has been in preparation for some time. We hope, from what we know of these proposals, that they will secure hospital accommodation of the best kind for members of the middle and lower classes for an inclusive payment of from two to three guineas per week. The proposal to erect one large pay hospital in the neighbourhood of Cavendish Square for the accommodation of paying patients cannot, in practice, provide the necessary accommodation at rates which will be within the means of the patients who most need such accommodation, and for this reason, whatever else it might accomplish, it would certainly not solve the problem raised by the recent correspondence.

